# Toxicokinetics and Perfluorooctanesulfonic Acid-Induced Liver Protein Expression Are Markedly Altered in Mice Lacking Albumin

**DOI:** 10.1021/acs.chemrestox.4c00508

**Published:** 2025-06-10

**Authors:** Emily M. Kaye, Jitka Becanova, Simon Vojta, Rainer Lohmann, Fabian Christoph Fischer, Angela Slitt

**Affiliations:** † Department of Biomedical and Pharmaceutical Sciences, College of Pharmacy, 4260 University of Rhode Island, 7 Greenhouse Rd, Kingston, Rhode Island 02881, United States; ‡ Graduate School of Oceanography, University of Rhode Island, Narragansett, Rhode Island 02882, United States; § Harvard John A. Paulson School of Engineering and Applied Sciences, Harvard University, Cambridge, Massachusetts 02138, United States

## Abstract

Perfluorooctanesulfonic acid (PFOS) is a ubiquitous perfluoroalkyl substance (PFAS) linked to liver disease and obesity in humans. Binding studies suggest that albumin is a crucial blood protein influencing PFOS toxicokinetics and hepatotoxicity; however, its role has not been mechanistically tested in vivo. This study used an albumin-deficient mouse model to investigate the relevance of albumin in PFOS tissue distribution and liver disease end points. Adult male C57BL/6J wild-type (Alb^+/+^) and albumin-deficient (Alb^–/–^) mice were orally gavaged daily for 7 days with either vehicle or PFOS at 0.5 or 10 mg/kg body weight. The measured PFOS concentrations in plasma were significantly lower in Alb^–/–^ mice compared to those in Alb^+/+^ mice, while liver concentrations were significantly higher in Alb^–/–^ mice. Binding experiments confirmed these findings, indicating that PFOS toxicokinetics are driven by plasma and tissue binding. Significant changes in liver protein expression did not translate into differences in liver disease end points between genotypes, suggesting the need for chronic exposure studies. Our data imply that disease-related albumin deficiency in humans can influence PFAS toxicokinetics and susceptibility to hepatotoxicity. Our framework using knockout mice can be adapted in future studies to assess the relevance of protein binding and membrane transporters in PFAS distribution and elimination.

## Graphical abstract

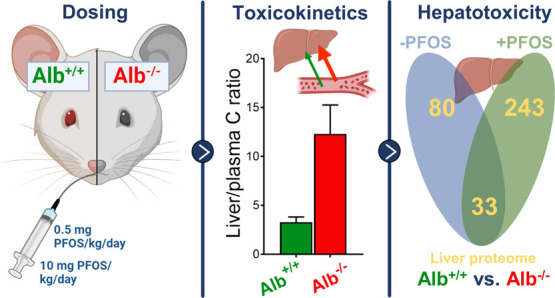

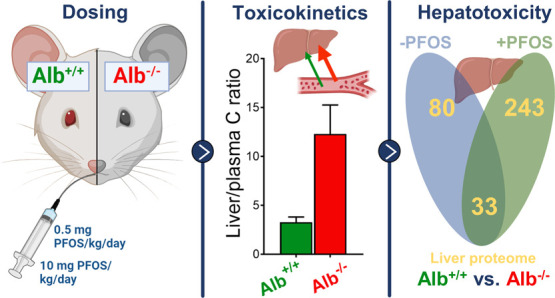

## 1 Introduction

Perfluoroalkyl substances (PFAS) are a diverse class of man-made environmental chemical pollutants. Human epidemiological data have associated PFAS exposure with kidney disease, modified thyroid function, altered developmental and reproductive function, and cancer.^1^ Perfluorooctanesulfonic acid (PFOS) is a legacy PFAS in this group of chemicals with a long elimination half-life in humans ranging between 3.3 and 27 years.^2,3^ Despite phase-out efforts, human biomonitoring studies have consistently detected PFOS in human serum worldwide,^4–7^ with average concentrations of ∼4 μg/L in the U.S. in 2018.^4,5^ PFOS exposures in humans have been linked to liver injury, dyslipidemia, and obesity in humans.^8–10^ Several studies have suggested that binding to blood proteins is a major factor in the slow elimination and subsequent chronic hepatotoxicity of PFOS in humans.^11,12^ However, this mechanism has not been investigated in vivo, which is the focus of this work.

PFOS administration increased serum liver enzymes, cytotoxicity, and steatosis in mice,^8^ rats,^13^ and monkeys.^14^ Epidemiological studies have found associations with PFOS exposure and elevated serum liver function tests,^15,16^ elevated serum cholesterol,^17^ and steatosis in humans.^18^ PFOS has been shown to activate multiple nuclear receptors (e.g., peroxisome proliferator-activated receptor alpha [PPARα]) and metabolic pathways in both rodent and primary human hepatocytes.^19^ A recent meta-analysis involving 24 human studies found associations between human PFOS exposure, higher alanine transaminase (ALT) levels (*z*-score = 3.55, *p* < 0.001), and liver damage.^20^ However, such studies have rarely established relationships between systemic PFOS exposures, liver accumulation, and associated hepatotoxicity.

Binding to blood proteins is thought to be a significant factor in determining systemic exposures and target site concentrations of PFOS in humans.^11^ Specifically, studies employing equilibrium dialysis and solid-phase microextraction (SPME) have demonstrated that PFAS with long fluorinated carbon chains like PFOS (8 fluorinated carbons) strongly binds to human serum albumin (HSA),^21,22^ the most abundant protein in human blood. Based on these studies, HSA binding is considered a major driver for PFOS toxicokinetics in humans.^11^ Still, the relative contribution of albumin binding to the distribution, elimination, and hepatotoxicity of PFOS under complex physiological conditions in vivo has not been investigated.

Plasma protein binding is a well-established mechanism influencing the half-life of pharmaceuticals.^23^ Molecules that bind poorly to plasma proteins are more readily available to penetrate cellular membranes and are more likely to be eliminated through the kidneys.^24^ PFOS primarily accumulates in the noncellular blood fraction,^25^ where albumin is the most abundant PFOS ligand,^21,26^ contributing 3.5–5 g/dL (50–60% of total proteins) to plasma in healthy humans.^27,28^ Being essential for homeostasis of oncotic pressure^29^ and as a carrier for molecules like fatty acids,^30^ albumin is secreted into the portal circulation after synthesis in the liver.^31^ Numerous binding studies have demonstrated that PFOS binds to BSA and HSA with a high affinity;^21,32–34^ however, the relative importance of albumin binding to other toxicokinetic processes like permeability,^35,36^ membrane transporter interactions,^37,38^ reabsorption,^39,40^ and elimination pathways present under realistic exposure conditions in vivo is unknown.

In this study, PFOS was dosed to a homozygous albumin knockout mouse model (C57BL/6J, Alb^–/–^) to investigate the relevance of albumin binding to PFOS plasma concentration, tissue accumulation, urine levels, and biomarkers associated with liver toxicity. See Section S1 of the Supporting Information for a schematic representation of the study design. The results were compared with those of wild-type mice (C57BL/6J, Alb^+/+^) to test the effects of albumin deficiency. To determine the extent to which PFOS distribution results from tissue binding, we compared the in vivo results with tissue distribution patterns measured using a previously established binding assay with C18 SPME fibers.^21^ We discuss the applicability of knockout mouse models for mechanistic studies on PFAS exposures and effects. Finally, we highlight the implications of our findings in the context of disease-associated albumin deficiency in humans, the resulting susceptibility to PFAS liver accumulation, and the associated health effects.

## 2 Materials and Methods

### 2.1 Chemicals and Reagents

PFOS was purchased from Sigma-Aldrich as a heptadecafluoro-octanesulfonic acid potassium salt (CAS: #2795–39–3, Catalog: #89374, ≥98.0% purity, ∼ 70% L-PFOS/∼30% Br-PFOS). For C18 fiber binding experiments, the PFAC-24PAR mixture from Wellington Laboratories was used, which includes carboxylates, sulfonates, fluorotelomers, and sulfonamides. The corresponding mass-labeled standard PFAS mixture from Wellington Laboratories was used as an internal standard. Iodoacetamide (IAA), sodium deoxycholate (NaDOC), dithiothreitol (DTT), formic acid, chloroform, methanol, acetonitrile, urea, ammonium bicarbonate, Tween 20, and bovine serum albumin (BSA) were purchased from Sigma-Aldrich. Trypsin (TPCK-treated trypsin) was purchased from SCIEX. Primers for β-actin, Ehhadh, Acot2, cluster of differentiation 36 (Cd36), fatty acid binding protein 1 (Fabp1), fatty acid binding protein 4 (Fabp4), Cyp27a1, and Fatp2 were purchased from Invitrogen.

### 2.2 Treatment Paradigm and Dosing Solutions

All animal protocols were reviewed and approved by the University of Rhode Island (URI) Institutional Animal Care and Use Committee (IACUC). The study overview is illustrated in Figure S1. Alb^–/–^ mice were purchased from Jackson Laboratory. Genotypes of Alb^–/–^ and wild-type (WT) mice were confirmed by PCR either in-house or by The Jackson Laboratory. The Alb^–/–^ model has been previously validated to lack albumin expression.^41^ Briefly, a transcription activator-like effector nuclease (TALEN)-mediated nonhomologous end-joining (NHEJ) repair pathway was utilized to knock out the albumin gene in C57BL/6J mice. TALEN mRNAs were introduced into C57BL/6J zygotes and crossed with a WT mouse. The F1 generation was then crossed to produce Alb^–/–^ homozygotes. Upon arrival, the mice were randomly assigned to treatment groups and were housed in a temperature-controlled room (20–26 °C) with relative humidity (30–70%) and lighting (12 h, light–dark cycles) and fed *ad libitum* a standard chow diet (Harlan Teklad Extruded Global Diet). Male WT C57BL/6J and Alb^–/–^ mice were dosed daily via oral gavage with PFOS at either 0.5 mg/kg_bw_ (bw, body weight) (9 weeks old, *n* = 6) or 10 mg/kg_bw_ (19 weeks old, *n* = 4) for 7 days. Although different in age, both cohorts fell within the adult range for mice, and the 9 week age group aligns with prior PFOS toxicity studies.^42^ The 10 mg/kg_bw_ group included four mice per genotype due to limited colony availability, while six mice per genotype were used in the 0.5 mg/kg_bw_ group. PFOS was dissolved in 0.5% Tween 20 in Millipore-treated water, yielding a final dosing volume of 10 mL/kg_bw_. PFOS-free 0.5% Tween 20 in Millipore-treated water was dosed as a vehicle control (VEH). The 10 mg/kg_bw_ dose was selected based on prior rodent studies, demonstrating robust liver responses after 7 days of repeated dosing, including hepatomegaly, steatosis, and activation of lipid metabolism pathways such as PPARα.^42^ This dose is above human environmental exposure levels but was used here to induce a measurable liver response within a short exposure duration and to evaluate albumin’s role under conditions of known PFOS hepatotoxicity. The lower 0.5 mg/kg_bw_ dose, while still higher than typical general population exposures, was included to assess albumin’s influence under subtoxic conditions^42^ more relevant to upper-bound environmental or occupational exposures.

### 2.3 Clinical Chemistry

Plasma was collected at the time of necropsy and stored at −80 °C until analysis. Plasma alanine aminotransferase (ALT) activity was determined using the colorimetric ALT activity assay kit (Pointe Scientific Inc.). Triglycerides were quantified from plasma using a triglyceride (GPO) reagent colorimetric assay kit (Pointe Scientific Inc.). Free fatty acids were measured from plasma by using a free fatty acid assay kit (Abcam). Creatinine in urine was measured using a mouse creatinine assay kit from Crystal Chem (Elk Grove Village).

### 2.4 Liver Lipid Isolation

Images of the sampled livers can be found in Section S2 of the Supporting Information. Total liver lipids were isolated using the adapted chloroform/methanol Folch method.^43^ Briefly, ∼50 mg of liver tissue was homogenized in 1 mL of methanol using an Omni Bead Ruptor Elite system (Omni International) for 30 s at 5 m/s. After homogenization, an additional 1 mL of methanol was added. The total water content of each liver sample (65%) was calculated, and the volume was adjusted to 1 mL with water. Subsequently, 900 μL of chloroform was added, and the mixture was vortexed. Then, 1 mL of water and another 900 μL of chloroform was added, vortexed, and centrifuged at 1200 rpm for 10 min for phase separation. Total lipid mass was determined by transferring the bottom organic layer to a preweighed glass tube, evaporated under nitrogen, and weighed.

### 2.5 PFOS Extraction from Plasma and Tissues

Liquid chromatography–mass spectrometry (LC–MS/MS) was used to quantify PFOS concentrations in plasma and tissues, using a X500R QTOF platform coupled to an ExionLC AC System (SCIEX), as published previously.^44^ Instrumental method details are given in Section S3. Snap-frozen tissues were cut into ∼22 mg pieces, and 5 μL of plasma was used as input and spiked with 100 ng of a C4-PFOS-labeled internal standard (Wellington Laboratories). All samples were processed alongside procedural blanks and replicates, and PFOS was quantified by using matrix-matched calibration curves. Tissues were homogenized in 440 μL 50:50 acetonitrile/water using the OMNI International Bead Ruptor Elite bead mill homogenizer at 5 m/s for 30 s. Plasma was vortexed with the PFOS internal standard in 100 μL of 50:50 acetonitrile/water until thoroughly mixed. PFOS was isolated from tissues and plasma utilizing the roQ QuEChERS Extraction Packet and dSPE kits (Phenomenex), with protocol modifications for working in smaller volumes. Briefly, for tissues, 110 mg of the roQ QuEChERS Extraction Packet (4.0 g of magnesium sulfate +1.0 g of sodium chloride) was added to the homogenized tissue, shaken, vortexed for 15 s, and centrifuged at 4000*g* for 16 min for phase separation. The top 150 μL was then taken and added into a tube containing 30 mg of dSPE powder (150 mg of magnesium sulfate +50 mg of primary secondary amine), shaken, vortexed (15 s), and centrifuged (4000*g*/16 min). The top 75 μL was sampled and measured by LC–MS/MS. For plasma, 25 mg of the roQ QuEChERS Extraction Packet was added to the plasma, shaken, vortexed (15 s), and centrifuged (4000*g*/16 min). The top 45 μL was then taken and added into a tube with 9 mg of dSPE powder, shaken, vortexed (15 s), and centrifuged (4000*g*/16 min). The top 30 μL was sampled and measured by LC–MS/MS. The 10 mg/kg_bw_ tissues and plasma were diluted 400×, and the 0.5 mg/kg_bw_ tissues and plasma were diluted 20× before measurements.

### 2.6 C18 Fiber Solid-Phase Microextraction

Binding of 24 PFAS to mouse plasma and liver was measured using a previously developed C18 fiber-based SPME technique. Plasma and liver samples from PFOS-free vehicle control Alb^+/+^ and Alb^–/–^ mice were used for the experiments (*n* = 3). Details on methodologies are published elsewhere.^21^ In short, C18 SPME fibers were preconditioned and placed into PFAS-spiked (400 ng/L) mouse plasma or tissue homogenates dissolved in phosphate-buffered saline (PBS). Additionally, C18 fibers were placed in a PFAS-spiked PBS buffer (400 ng/L). The fibers were incubated in the solution at 37 °C and 100 rpm horizonal shaking for 48 h and then extracted with methanol overnight. The difference in chemical mass sampled by the SPME fiber in the presence and absence of the tissue homogenate in solution is the chemical fraction bound to the tissue. Liver–water and plasma–water partition coefficients (*K*_liver/w_, *K*_plasma/w_) can be derived from this fraction and the mass of tissue in solution. Liver–plasma partition coefficients (*K*_liver/plasma_) which correspond to expected liver/plasma concentration ratios can then be calculated as

1 $$K_{liver/plasma}=\frac{K_{liver/w}}{K_{plasma/w}}=\frac{C_{liver}}{C_{plasma}}$$

### 2.7 Proteomics Sample Preparation and Analysis

Proteomic preparation and analysis were conducted by using snap-frozen livers from the 0.5 mg/kg_bw_ study. SWATH-DIA proteomics was conducted as previously published.^45^ In short, snap-frozen livers were homogenized in 8 M urea buffer, and the protein concentration was determined using the Pierce BCA Protein Assay Kit (catalog no. 23225) from ThermoFisher Scientific. Homogenates were diluted to 2 mg/mL protein/sample in 100 μL and spiked with 0.2 mg/mL BSA. Samples were denatured with DTT in a shaking water bath (100 rpm) for 15 min. Following denaturation, cysteine residues were reduced to the sulfhydryl form by incubating samples in the dark for 30 min with iodoacetamide (IAA). Milli-Q-purified water, ice-cold methanol, and ice-cold chloroform were added to precipitate the proteins and remove lipids and nucleic acids. Samples were centrifuged at 12,000 rpm for 5 min at 10 °C for phase separation and to pellet the protein between aqueous and organic layers. The supernatant was removed, and the pellet was subsequently washed gently with ice-cold methanol. The pellet was briefly dried before lysing the proteins by adding sodium deoxycholate (NaDOC) in 50 mM ammonium bicarbonate (3% w/v solution). Lyophilized trypsin was added to each sample, vortexed, and pipetted into MT-96 PCT MicroTubesTM (Pressure BioScience Inc.) before being placed in a Barocycler NEP2320 PCT Sample Preparation System (Source Scientific). A Haake SC 100 (ThermoFisher) water bath was utilized to bring the water up to the temperature for use in the Barocycler. The Barocycler program was: time 1 = 50 s, time 2 = 10 s, pressure = 35 psi, temp = 33 °C, cycles = 75 (run one) and 60 (run two). The samples were subjected to the Barocycler for run one, and lyophilized trypsin was added again before the samples were subjected to the Barocycler for the second run. After the runs were completed, 5% formic acid was added to each sample and centrifuged at 12,000 rpm for 10 min at 10 °C. After centrifugation, 75–100 μL of the supernatant was carefully removed and transferred into HPLC vials for analysis. A SCIEX 5600 TripleTOF mass spectrometer in positive electrospray ionization mode equipped with a DuoSpray ion source (SCIEX) coupled to an Acquity UHPLC HClass system (Waters Corp.) was used for measurements. Spectronaut (Biognosys) was used to process the resulting data files from the SWATH-MS runs with a reference spectral library from the UP000000589_mice reference protein database. The total protein approach was used to convert raw intensities to pmol/mg of protein. The mass spectrometry proteomics data have been deposited to the ProteomeXchange Consortium (http://proteomecentral.proteomexchange.org) via the PRIDE partner repository.^46^

### 2.8 Gene Expression by RT-qPCR

Livers snap-frozen at the time of necropsy were cut into 15–25 mg pieces and prepared using the IBI Total RNA Mini Kit (IBI Scientific). RNA was quantified using a ThermoFisher Nanodrop 1000 and diluted with diethyl pyrocarbonate (DEPC) water to equal concentrations. Complementary DNA (cDNA) was synthesized using the High-Capacity cDNA Reverse Transcription Kit (ThermoFisher) and run on a thermal cycler (Eppendorf) at 25 °C for 10 min, 37 °C for 120 min, 85 °C for 5 min, and finally a hold at 4 °C indefinitely. RT-qPCR was performed using specific primers for β-actin, Ehhadh, Acot2, Cd36, Fabp1, Fabp4, Cyp27a1, and Fatp2. These target genes were selected based on their established role as prototypical PFOS-responsive transcripts in the liver, independent of the proteomic analysis.

### 2.9 Statistics

A two-way mixed analysis of variance (ANOVA) was used for statistical data analysis to consider for both within-replicate (tissue concentrations, biomarker levels) and between-treatment effects (Alb^+/+^ vs Alb^–/–^ mice, vehicle vs PFOS-dosed). This statistical approach was chosen to address the sampling and quantification of multiple tissues and biomarkers for individual mice. The ANOVA assumes uniform variability among differences across all paired conditions. To further assess statistically significant differences in tissue concentrations and biomarker levels between the mouse genotypes (Alb^+/+^ vs Alb^–/–^ mice), dosage forms (PFOS vs vehicle), and dosages (0.5 vs 10 mg PFOS/kg_bw_/day), Šidák’s multiple comparison tests were performed with a significance threshold of *p* < 0.05. Statistical tests were run in GraphPad prism (version 10.1.2). The *p* values are reported in the plots throughout the manuscript.

## 3 Results and Discussion

### 3.1 PFOS Concentrations in Plasma and Organs

PFOS concentrations in mice organs generally followed the trend liver > lung > kidney > intestines for both the dosing regimens and genotypes (Figure 1). In albumin-deficient mice (Alb^–/–^), liver concentrations were 1.25-fold (10 mg/kg_bw_/day, *p* = 0.02) and 1.14-fold (0.5 mg/kg_bw_/day, *p* = 0.04) higher than those in wild-type (Alb^+/+^) mice. The liver accounted for 28.9% ± 2.6% and 35.7% ± 2.0% of the total administered dose in Alb^+/+^ and Alb^–/–^ mice, respectively, at 10 mg/kg_bw_/day. At 0.5 mg/kg_bw_/day, the liver retained 24.4% ± 4.5% in Alb^+/+^ and 31.5% ± 3.7% in Alb^–/–^ mice, demonstrating consistently higher hepatic PFOS accumulation in albumin-deficient mice across both exposure levels. Conversely, plasma concentrations were reduced by 65% (10 mg/kg_bw_/day) and 52% (0.5 mg/kg_bw_/day) in Alb^–/–^ as compared to Alb^+/+^ mice (*p* < 0.01). Kidney concentrations were slightly higher in Alb^–/–^ mice (mean: 75 μg/g) compared to those in Alb^+/+^ mice (mean: 71 μg/g) at 10 mg/kg_bw_/day, though this difference was not statistically significant. At the lower 0.5 mg/kg bw/day dose, a statistically significant difference was observed, with Alb^–/–^ mice exhibiting 1.2-fold higher kidney PFOS levels than wild-type controls (*p* = 0.01). Interestingly, PFOS levels in urine were significantly reduced in Alb^–/–^ mice exposed to 10 mg PFOS/kg_bw_/day as compared to Alb^+/+^ mice (Figure S3), indicating impaired tubular secretion due to reduced uptake into proximal tubule cells, as albumin has been shown to facilitate organic anion transporter-mediated transport of protein-bound compounds.^47^

**Figure 1:**
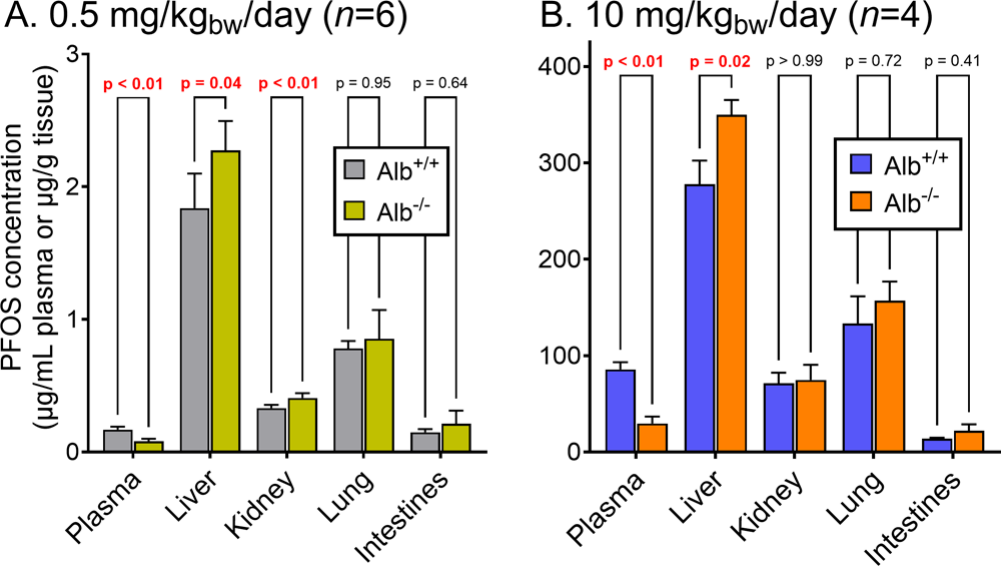
PFOS concentrations in plasma and various tissues of wild-type (Alb^+/+^) and albumin knockout (Alb^–/–^) mice exposed daily to 0.5 mg/kg_bw_ (A) or a 10 mg/kg_bw_ (B) PFOS for 7 days. Data were statistically analyzed using a two-way mixed ANOVA, followed by a Šidák post hoc test. The *p* values for multiple comparisons are reported in the graph, and significant differences are highlighted in red (*p* < 0.05).

The strong reduction in plasma PFOS concentrations in Alb^–/–^ mice at both dose levels highlights the critical role of albumin for systemic PFOS exposures, confirming previous in vitro and binding studies that demonstrated the strong binding of PFOS to isolated albumin proteins.^21,32,33,48^ The residual plasma PFOS in Alb^–/–^ mice suggests that other plasma components bind PFOS to a considerable extent. A recent binding study showed that globulins bind long-chain PFAS including PFOS,^21^ which could explain the residual plasma concentrations observed here. While the PFOS levels in plasma decreased, 14–25% increased concentrations were measured in the liver of Alb^–/–^ mice compared to Alb^+/+^ mice. Contrarily, PFOS concentrations were not statistically elevated in the lungs and intestines of Alb^–/–^ mice. A possible explanation is that PFOS transport into liver tissue could be facilitated by organic anion transporter proteins (OATPs), as shown in in vitro cell assays.^49^ To investigate the relevance of facilitated transport indirectly, tissue binding experiments were conducted using PFAS-spiked liver and plasma from vehicle control Alb^–/–^ and Alb^+/+^ mice, and the results were compared to in vivo observations.

### 3.2 Tissue Binding Experiments Confirm In Vivo Observations

The binding experiments with plasma and liver tissues yielded liver–plasma partition coefficients (*K*_liver/plasma_) Alb^–/–^ and Alb^+/+^ mouse tissues, corresponding to the expected liver/plasma concentration ratios, assuming binding as the sole mechanism for PFAS distribution (eq 1). For PFOS, binding experiments predicted a 2.9-fold increase in liver/plasma concentration ratios in Alb^–/–^ mice compared to Alb^+/+^ mice (Figures 2 and S4). This prediction aligns well with in vivo observations, where 3.8-fold and 2.7-fold increase were observed for the 10 mg/kg_bw_/day and 0.5 mg/kg_bw_/day treatments, respectively.

**Figure 2:**
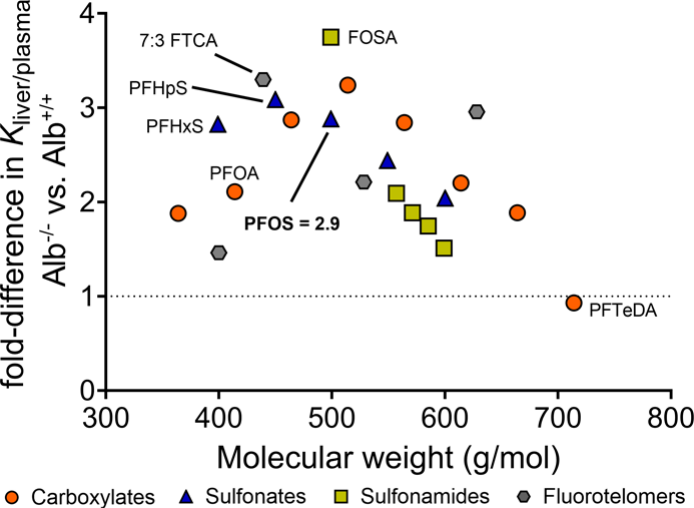
Fold-difference in liver–plasma partition coefficients measured for liver and plasma tissues sampled from Alb^–/–^ mice as compared to Alb^+/+^ mice. PFAS classes are indicated by the color and shape of symbols, with orange circles for carboxylates, blue triangles for sulfonates, yellow squares for sulfonamides, and gray hexagons for fluorotelomers.

The increased liver concentrations observed in Alb^–/–^ mice could result from higher levels of unbound PFOS in the plasma as less PFAS are bound to albumin.^24^ Essentially, with reduced albumin, the plasma’s capacity to retain PFOS reduces, leading to more extensive PFOS transport to the highly perfused liver and kidneys. This assumption is supported by our binding experiments, which showed a 3.5-fold increase in unbound fractions of PFOS in Alb^–/–^ mice as compared to Alb^+/+^ mice (Figure S5A). Interestingly, a 1.2-fold increase in unbound fractions of PFOS in the livers of Alb^–/–^ mice as compared to Alb^+/+^ mice was measured (Figure S5B), indicating the presence of albumin in rodent livers that contributes to PFOS binding. This finding is consistent with the liver being the site of albumin synthesis.^31^

The liver/plasma distribution of perfluorooctane sulfonamide (FOSA) and 7:3 fluorotelomer carboxylic acid (7:3 FTCA) was most strongly influenced by albumin binding (Figure 2), suggesting to prioritize these compounds in future experiments with albumin knockout mice. The relative importance of albumin binding decreases with an increase in molecular weight for PFAS carboxylates, sulfonates, and sulfonamides (Figures 2 and S4). Notably, an opposite trend was observed for fluorotelomers. The C14 perfluorotetradecanoic acid (PFTeDA) showed similar binding to Alb^–/–^ and Alb^+/+^ plasma and livers (Figure S6). PFAS with more than 12 fluorinated carbon atoms exhibit low binding to albumin,^21^ which could explain why PFTeDA distribution was not affected by albumin deficiency. The agreement between mouse experiments and binding assays suggests that PFOS liver/plasma distribution is primarily influenced by tissue binding instead of kinetic processes like permeability^35,36^ or membrane transporters (e.g., OATPs).^38^ Shorter chain PFAS like perfluorohexanesulfonic acid (PFHxS, CF6) demonstrate low permeability,^35^ strong associations with membrane transporters,^35^ high albumin binding,^21^ and distribution to both liver and plasma (Figure S6). Future in vivo studies with knockout mice could reveal the relative importance of these toxicokinetic processes to the accumulation of these shorter-chain PFAS in target organs like the liver.

### 3.3 Changes in Total Body and Liver Weights

The body weights of both Alb^+/+^ and Alb^–/–^ mice were not significantly different between vehicle controls and PFOS-dosed mice for both dosage groups (Figure S7). This observation is consistent with a previous study that demonstrated no effects on body weight at a daily dose of 10 mg PFOS/kg_bw_.^42^ In both Alb^+/+^ and Alb^–/–^ mice exposed to 10 mg of PFOS/kg_bw_/day, liver weights significantly increased compared to vehicle controls (Figure 3A). Specifically, liver-to-body weight ratios of Alb^+/+^ mice dosed daily with 10 mg/kg_bw_ of PFOS were 1.8-fold higher than for the livers of vehicle controls (*p* < 0.01). Similarly, PFOS-dosed Alb^–/–^ mice showed a 2.3-fold increase in liver weight compared to vehicle controls (*p* < 0.01). No significant differences in liver-to-BW ratios were observed among 0.5 mg of PFOS/kg_bw_/day-dosed and PFOS-free Alb^+/+^ and Alb^–/–^ mice (Figure 3A).

**Figure 3:**
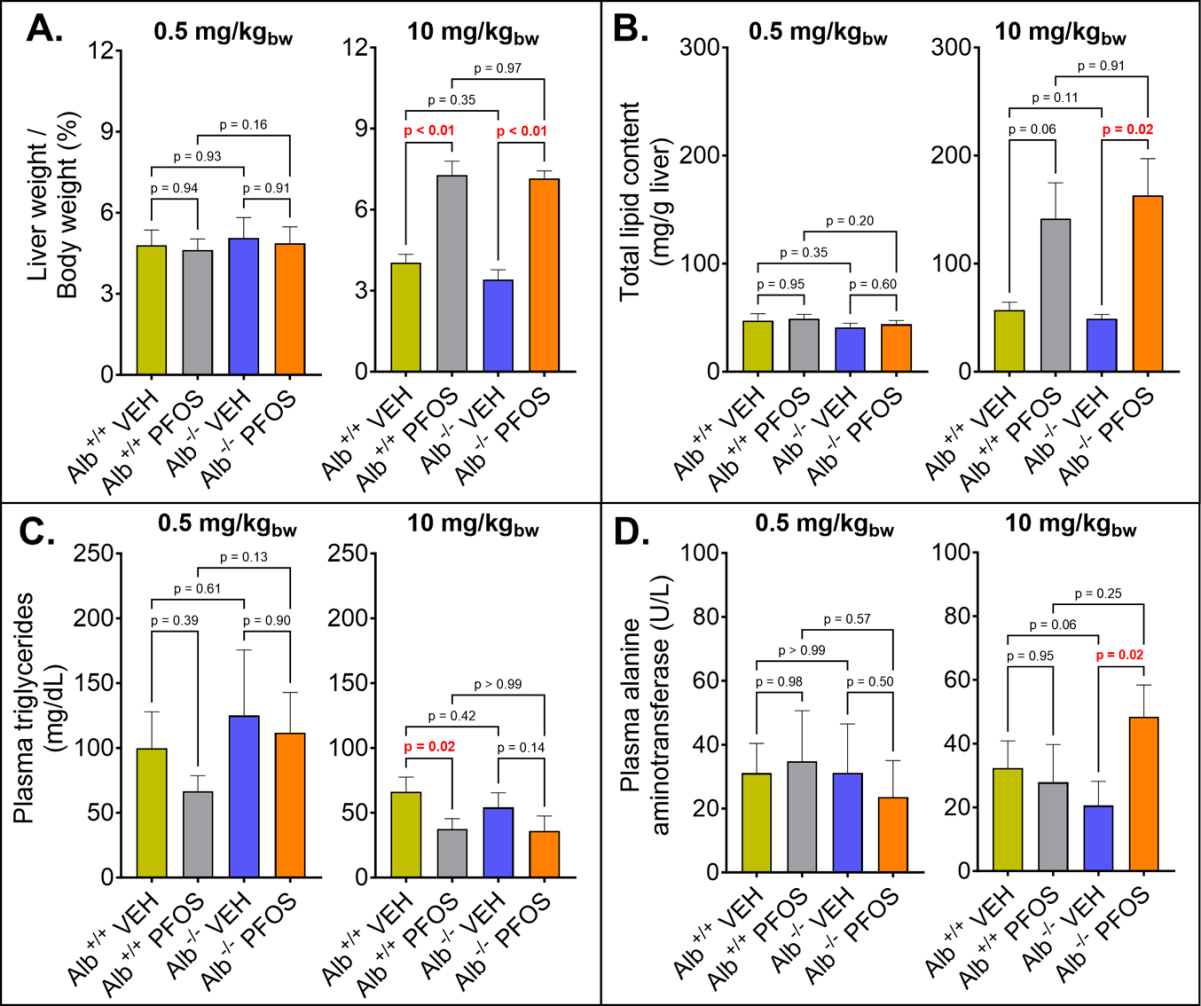
Liver weight to body weight ratios (A), total liver lipid contents (B), plasma triglyceride levels (C), and plasma alanine aminotransferase levels (D) of wild-type (Alb^+/+^) and albumin knockout (Alb^–/–^) mice exposed daily to PFOS-free vehicle (VEH) or a daily dose of 0.5 mg/kg_bw_ or 10 mg/kg_bw_ PFOS for 7 days. Data were statistically analyzed using a two-way mixed ANOVA, followed by a Šidák post hoc test. The *p* values for multiple comparisons are reported in the graph, and significant differences are highlighted in red (*p* < 0.05).

The observed liver enlargement at high PFOS exposures is consistent with previous rodent studies^42^ and is likely caused by the increased proliferation of hepatocyte peroxisomes.^15^ Despite the significantly higher PFOS concentrations (14–25%) observed in the liver of Alb^–/–^ mice compared to Alb^+/+^ mice (Figure 1), there was no corresponding increase in liver size between PFOS-dosed Alb^+/+^ and Alb^–/–^ mice (Figure 3A). This suggests that the mechanisms responsible for PFOS-induced liver enlargement are independent of the elevated PFOS liver concentrations observed in albumin-deficient mice within the context of a 7 day PFOS exposure.

### 3.4 Liver Lipid Content and Triglyceride Levels

Liver lipids and plasma triglyceride levels were analyzed to explore the role of albumin in lipid regulation in mice administered daily doses of 0.5 or 10 mg of PFOS/kg_bw_. In the 0.5 mg/kg_bw_ dosage group, no significant differences in liver lipid content were observed either within or between PFOS-dosed Alb^+/+^ and Alb^–/–^ mice (Figure 3B). For the 10 mg/kg_bw_ dosage, Alb^+/+^ mice exhibited a 1.5-fold increase in liver lipid content compared to vehicle controls (*p* < 0.01). Similarly, Alb^–/–^ mice treated with PFOS displayed a 2.3-fold increase in liver lipid levels compared to their VEH controls (*p* < 0.01). There was no significant difference in the lipid content between PFOS-dosed Alb^+/+^ and Alb^–/–^ mice for both doses. These findings agree with the total liver weights and liver/body weight ratios (Figure 3A), further indicating that albumin does not play a role in the changes of lipid deposition over a 7 day PFOS dose. In the 0.5 mg/kg_bw_ daily dosage group, there were no significant differences in plasma triglyceride (TG) levels among PFOS-dosed and vehicle control Alb^+/+^ and Alb^–/–^ mice (Figure 3C, left plot). Conversely, Alb^+/+^ mice dosed daily with 10 mg of PFOS/kg_bw_ exhibited a significant 43% decrease in plasma TG levels (*p* = 0.02). Similarly, plasma TG levels in 10 mg PFOS/kg_bw_-dosed Alb^–/–^ mice reduced to 66% of the levels observed in the corresponding vehicle controls; however, these were nonsignificant trends (*p* = 0.14).

### 3.5 Alanine Aminotransferase Levels

ALT levels were assessed as biomarkers for liver damage. In the 0.5 mg/kg_bw_ dosage group, differences in plasma ALT levels were not statistically significant among PFOS-dosed and vehicle-dosed Alb^+/+^ and Alb^–/–^ mice (Figure 3D, left plot). For the 10 mg/kg_bw_ dosage, while plasma ALT levels in Alb^+/+^ mice showed a slight decrease, they did not significantly differ from those in the control group (*p* = 0.25). However, Alb^–/–^ mice treated with PFOS exhibited a 1.6-fold significant increase in plasma ALT levels (*p* = 0.02, Figure 3D, right plot). Plasma ALT data were generally inconsistent and not in line with previously published data;^8,15,42^ however, the increase in ALT levels is consistent with the observations of significantly increased liver weight and lipid accumulation (Figure 3A,B), suggesting liver damage caused by the daily 10 mg/kg_bw_ PFOS dose.

### 3.6 Changes in Liver Proteome

Proteomic analyses were conducted on liver samples from Alb^+/+^ and Alb^–/–^ mice that had been dosed daily with vehicle or 0.5 mg/kg_bw_ of PFOS, to explore the role of albumin on PFOS-induced alterations in liver protein expression under moderate exposure levels that did not trigger hepatotoxicity after 7 days (Figure 3). Protein levels differed considerably across all groups, suggesting that the baseline levels of proteins in Alb^–/–^ mice differ distinctly from those of Alb^+/+^ mice (WT), regardless of the PFOS exposure (Figure 4). However, the most significant proteomic changes were observed between PFOS-exposed Alb^+/+^ and Alb^–/–^ mice, indicating the important role of albumin in PFOS-induced alterations in liver protein expression. Specifically, in Alb^–/–^ mice exposed to PFOS, 195 proteins were uniquely altered compared to those in PFOS-exposed Alb^+/+^ mice (Figure 4), highlighting the significant role of albumin deficiency on the liver proteome response to PFOS.

**Figure 4:**
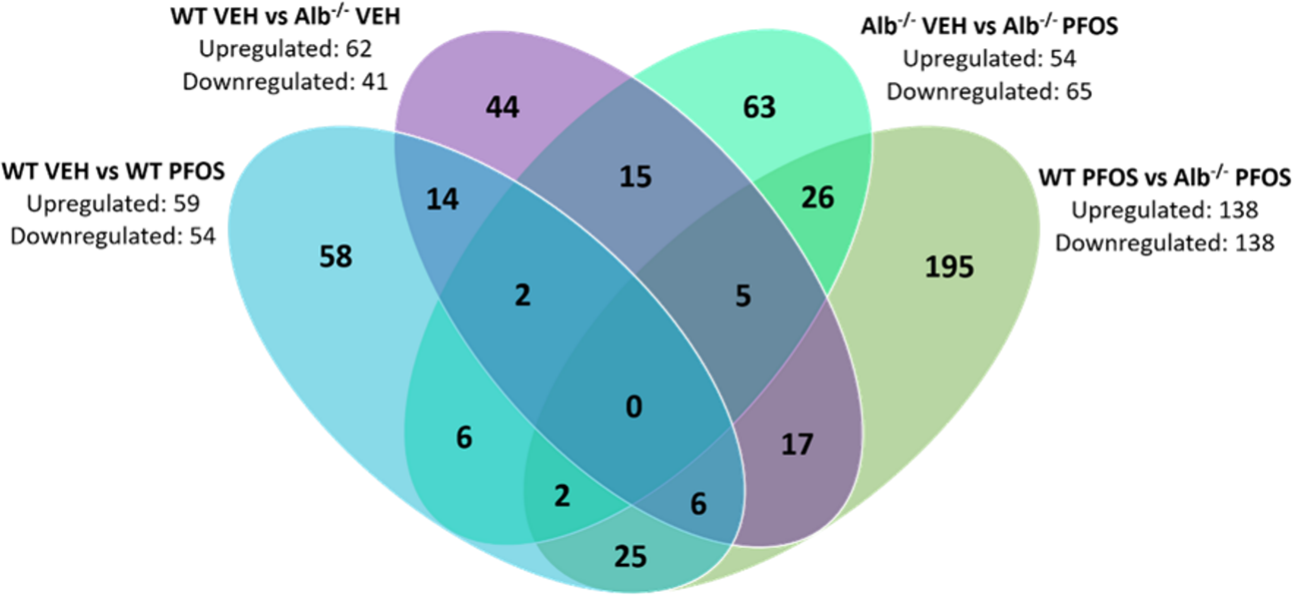
Individual and commonly observed proteins across treatment comparisons (WT = Alb^+/+^; VEH = vehicle control). The overlapping areas represent proteins with commonly altered expression across multiple comparisons, while nonoverlapping areas indicate proteins with expression changes unique to specific treatments. The Venn diagrams were created by Log2 transforming the treatment comparison fold changes and filtering out all insignificant Log2FC values (*p* < 0.05).

Ingenuity pathway analysis indicated a moderate inactivation of proteins involved in the hepatocyte nuclear factor 4 alpha (HNF4a) pathway in PFOS-dosed Alb^–/–^ mice compared to that in Alb^+/+^ mice. HNF4a is a crucial regulator of hepatocyte differentiation and the maintenance of liver functions, influencing bile acids, lipids, glucose, and drug metabolism.^50,51^ It is also implicated in the onset and progression of nonalcoholic fatty liver disease (NAFLD) and is notably reduced in nonalcoholic steatohepatitis (NASH) patients and rodent models.^52^

### 3.7 Gene Expression Specific to Lipid Metabolism

Expression levels of genes specific to lipid metabolism and accumulation were analyzed using RT-qPCR, including Fabp1, Fabp4, Enoyl-CoA hydratase, 3-hydroxyacyl CoA dehydrogenase (Ehhadh), Cd36, Acyl-CoA thioesterase 2 (Acot2), Cytochrome P450 27A1 (Cyp27a1), and fatty acid translocase 2 (Fatp2). At daily doses of 0.5 mg PFOS/kg_bw_, there were no significant changes in the expression of Fabp1, Fabp4, Ehhadh, Acot2, and Cyp27a1 among PFOS-dosed and vehicle control Alb^+/+^ and Alb^–/–^ mice (Figure S8). Notably, Cd36 was significantly upregulated by 12- and 21-fold in PFOS-exposed Alb^+/+^ and Alb^–/–^ mice, respectively (Figure S8D). Fatp2 was significantly upregulated 1.7-fold in PFOS-exposed Alb^+/+^ mice (Figure S8G). Larger changes were observed for the 10 mg/kg_bw_/day treatment group. In both Alb^+/+^ and Alb^–/–^ mice, significant upregulation was observed: Fabp1 increased 2.2-fold and 2.4-fold, Ehhadh increased 25-fold and 34-fold, Cd36 increased 12-fold and 21-fold, and Acot2 increased 3.5-fold and 3.4-fold, respectively (Figure S8). No significant changes were observed for Cyp27a1, Fabp4, or Fatp2 at either PFOS dose.

Few studies have explored the connection between PFAS exposure and the HNF4a pathway, though a recent study confirmed alterations in proteins and genes of this pathway with PFOA and PFOS exposure.^53^ Differences in biological process interaction networks were highlighted by ShinyGo 8.0 (Figure S10). In Alb^–/–^ mice exposed to PFOS, unique biological processes such as the generation of precursor metabolites and energy, cellular respiration, aerobic respiration, and dicarboxylic acid metabolism were identified (Figure S10B). These findings provide insights into the differential responses observed between Alb^+/+^ and Alb^–/–^ mice under PFOS exposure.

### 3.8 Implications for PFOS Toxicokinetics and Toxicity in Humans

The primary objective of this study was to assess the role of albumin in PFOS liver accumulation and downstream hepatotoxicity. Fischer et al. recently postulated that variabilities in blood protein levels among humans could lead to differences in PFAS accumulation in toxicological target organs.^21^ Our rodent study demonstrates that decreased albumin levels can lead to significantly higher accumulation of PFOS in liver and kidneys (Figure 1) and that higher unbound fractions of PFAS establish in these organs that are available to interact with toxicological targets (Figure S4). Similar trends are to be expected for PFAS of various classes with molecular weights of 400–600 g/mol (Figure S2). Future in vivo studies with albumin knockout mice should prioritize PFAS for which (i) albumin deficiency is expected to significantly affect liver/plasma distribution based on binding experiments (Figure 2) and (ii) membrane transporter-mediated hepatic uptake was observed in vitro, such as PFHxS and fluorotelomers.^35^ Although the higher dose was selected to induce liver effects within a short time frame, consistent differences in PFOS plasma and liver concentrations between genotypes at both dose levels, including the lower, subtoxic dose, demonstrate the influence of albumin on PFOS disposition. These results indicate that albumin may affect PFAS toxicokinetics even at subacute doses relevant to humans. Studies under chronic, low-dose conditions are needed to confirm the implications for human health.

Considering the variability in albumin concentrations among humans due to genetic, environmental, and longitudinal factors,^54^ our study provides strong evidence that these differences could affect PFAS accumulation in target tissues, potentially leading to variable susceptibility to PFAS toxicity among individuals. For example, diseases that reduce albumin levels in blood, such as NAFLD,^12^ could lead to increased accumulation of PFAS in already functionally impaired liver tissue. This accumulation could trigger a feedback effect, as PFAS-induced hepatotoxicity could further decrease the level of albumin synthesis in the liver. We recommend future epidemiological studies to investigate the relationship among blood albumin levels, PFAS accumulation in target organs, and associated health effects.

Our 7 day exposure study with rodents demonstrated that proteins and genes within the HNF4a pathway and those associated with lipid metabolism and liver damage were significantly altered in PFOS-exposed Alb^–/–^ mice as compared to Alb^+/+^ mice. Despite these molecular changes, no significant differences were observed in the body weight, liver weight, or liver-to-body weight ratios, suggesting that albumin does not contribute to PFOS-induced liver enlargement. However, these end points were assessed after only 7 days of PFOS exposure, and differences in apparent hepatotoxicity might emerge with longer exposures. To predict the effects in the context of human exposures to PFAS, we suggest evaluating the effect of albumin deficiency on toxicity outcomes in a chronic exposure setup to determine if changes in PFAS distribution lead to differences in toxicities under prolonged exposure conditions. Our framework, which includes binding assays, measurements of plasma and organ concentrations, and toxicological end points, effectively elucidates mechanisms linking PFAS exposures to toxicities and supports the prioritization of end points measured in epidemiological studies. Future studies can adapt this framework to test other factors influencing PFAS toxicokinetics and toxicity such as binding to other tissue constituents (e.g., globulins) or facilitated uptake by membrane transporters (e.g., OATPs).

## Supplementary Material



## Notes

Schematic of study design; images of sampled livers; LC–MS/MS method; urine concentrations; additional results of C18 fiber-based binding experiments with 24 PFAS; temporal changes in body weights; changes in lipid metabolism gene expression; altered liver pathways; and protein interaction networks (PDF)
