# Supplementary material for: Toxicokinetics and Perfluorooctanesulfonic Acid-Induced Liver Protein Expression Are Markedly Altered in Mice Lacking Albumin
Source: Chem Res Toxicol. 2025 Jun 10;38(7):1183–91. doi: 10.1021/acs.chemrestox.4c00508 (PMC12422536; doi:10.1021/acs.chemrestox.4c00508)
Supplement: Supplementary file 1 [file tx4c00508_si_001.pdf]

Supporting Information

Toxicokinetics and perfluorooctanesulfonic acid (PFOS)-induced liver protein expression are markedly altered in mice lacking albumin

Emily M. Kaye[1], Jitka Becanova[2], Simon Vojta[2], Rainer Lohmann[2], Fabian Christoph Fischer[1,3,], and Angela Slitt[1,*]*

[1] Department of Biomedical and Pharmaceutical Sciences, College of Pharmacy, University of Rhode Island, 7 Greenhouse Rd, Kingston, RI, 02881, USA

[2] Graduate School of Oceanography, University of Rhode Island, Narragansett, Rhode Island 02882, United States

[3] Harvard John A. Paulson School of Engineering and Applied Sciences, Harvard University, Cambridge, Massachusetts 02138, United States.

*Address correspondence to: angela_slitt@uri.edu, fabian.fischer@uri.edu

Content overview: 9 Pages, 10 Figures

Section	Contents	Page
S-1.	Schematic of study design.	S-3
S-2.	Images of sampled livers.	S-3
S-3.	LC-MS/MS method.	S-4
S-4.	Urine concentrations.	S-4
S-5.	Additional results of C18 fiber-based binding experiments with 24 PFAS.	S-5
S-6.	Temporal changes in body weights.	S-6
S-7.	Changes in lipid metabolism gene expression.	S-7
S-8.	Altered liver pathways.	S-8
S-9.	Protein interaction networks.	S-9

S-1. Schematic of study design.

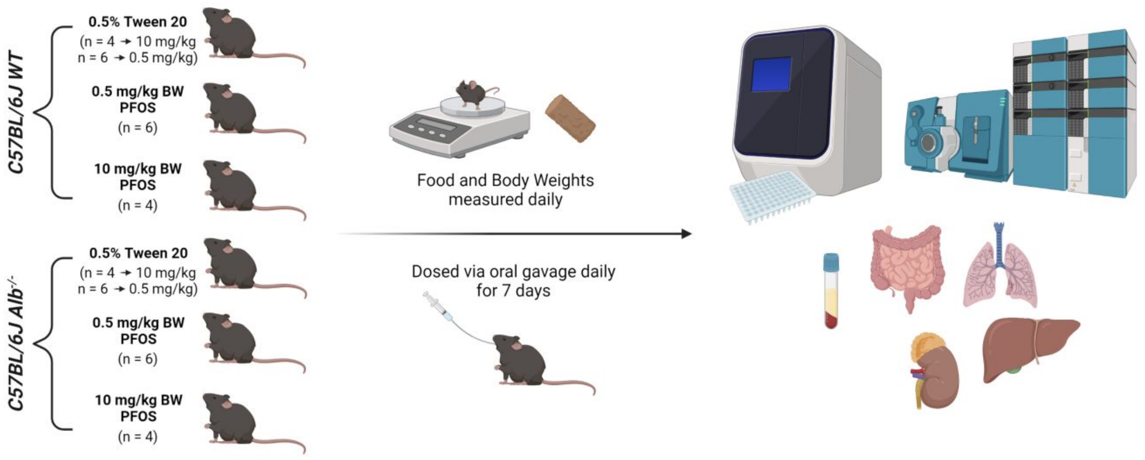

Figure S1. Schematic of study timeline, tissues extracted, and experiments performed. Created with Biorender.

S-2. Images of sampled livers.

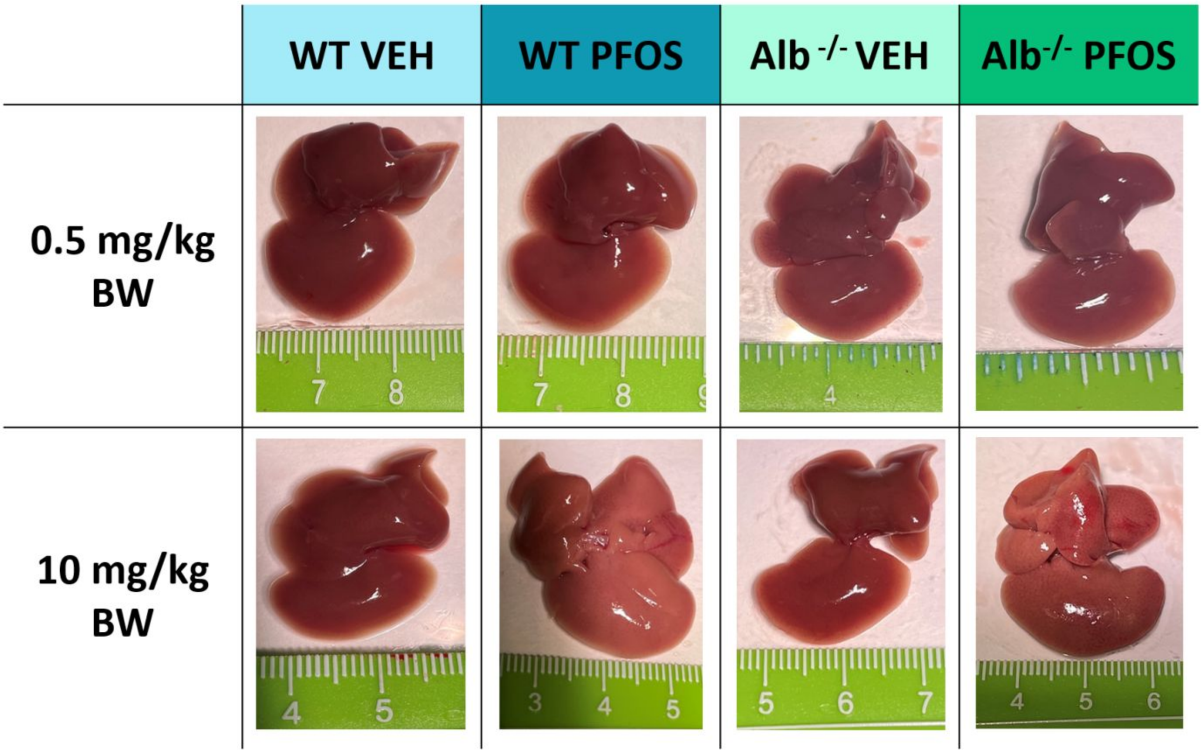

Figure S2. Livers at time of necropsy after daily dosing of either 0.5 mg/kg or 10mg/kg every day for 7 days. Treatment groups included: WT VEH (0.5% Tween 20), WT PFOS (0.5 or 10 mg/kg), $Alb^{-/-}$ VEH (0.5% Tween 20), $Alb^{-/-}$ PFOS (0.5 or 10 mg/kg). The 0.5 mg/kg dosed study had and n = 6/treatment group, the 10 mg/kg dosed study had and n = 4/treatment group.

S-3. LC-MS/MS method.

20 µL of the sample extract was injected on a Phenomenex Gemini C18 analytical column (3µm, 110Å, 50mm x 2mm) equipped with a Phenomenex SecurityGuard™ cartridge, at a flow rate of 0.3 mL min^{-1} and column temperature of 45 °C. A Phenomenex Gemini™ C18 analytical column (5 µm, 110 Å, 50mm x 4.6mm) was used as a delay column in order to avoid the PFAS instrumental contribution. The aqueous mobile phase (A) consisted of 10mM ammonium acetate in water and the organic mobile phase (B) was 10mM ammonium acetate in methanol. LC parameters were set to: 0.3 mL/min, 20 µL injection, column oven 45°C. The eluent gradient increased incrementally from 40% to 80% (1 to 5.5 min), 80% to 100% (5.5 to 7 min), held for one minute and then dropped to 40% (8 to 8.5 min) and held for another 6.5 min. For the quantification of the target analytes, a high-resolution tandem mass spectrometry (HRMSMS) method was used (MRM HR). Data were collected in negative electrospray ionization (ESI) mode using the following parameters: curtain gas pressure of 30 psi, ion source gas 1 at 40 psi, ion source gas 2 at 60 psi, and temperature of 450°C.

S-4. Urine concentrations.

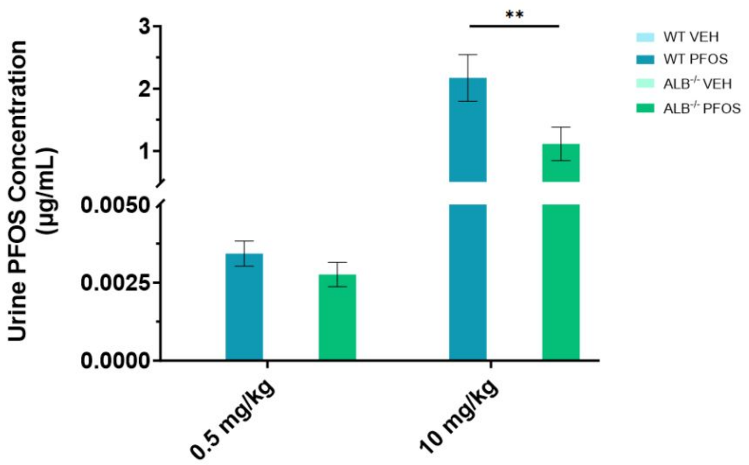

Figure S3. Urine concentrations measured in WT (Alb$^{+/+}$) and albumin-deficient (Alb$^{-/-}$) mice after 7 days of daily exposure to 0.5 mg or 10 mg of PFOS per kg body weight. Data were statistically analyzed using a two-way mixed analysis of variance (ANOVA) followed by a Šídák post-hoc test. Significant differences among the multiple comparisons are highlighted with asterisks.

S-5. Additional results of C18 fiber-based binding experiments with 24 PFAS.

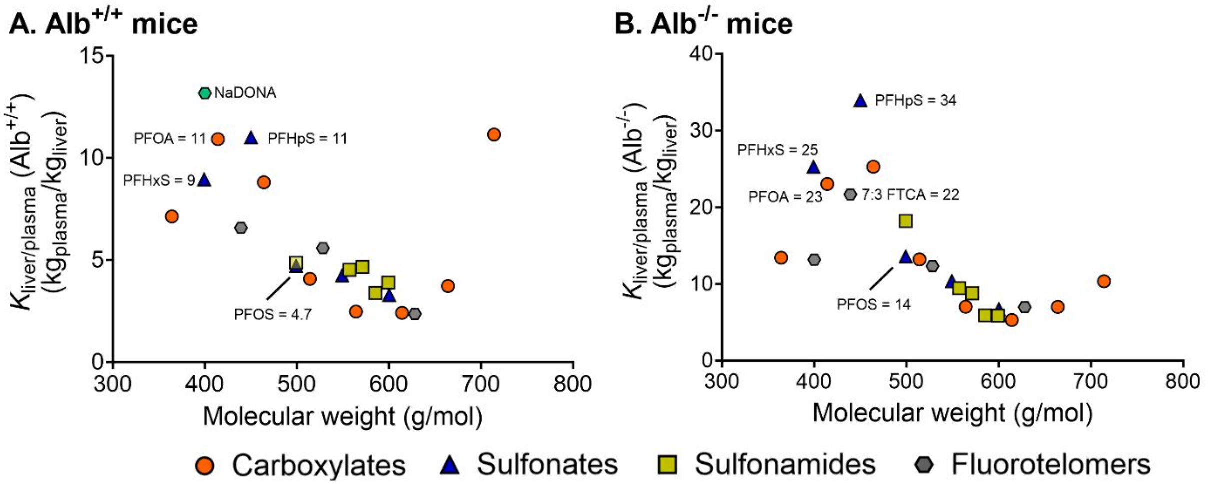

Figure S4. Liver-plasma partition coefficients ($K_{liver/plasma}$) as measured by C18 fiber-based binding experiments using tissues from vehicle-control Alb$^{+/+}$ (A.) and Alb$^{-/-}$ (B.) mice (n=3 per genotype). Please see section 2.6 of the main manuscript for method details. PFAS classes are indicated by the color and shape of symbols with orange circles for carboxylates, blue triangles for sulfonates, yellow squares for sulfonamides, and grey hexagons for fluorotelomers. Individual PFAS are highlighted by their abbreviations.

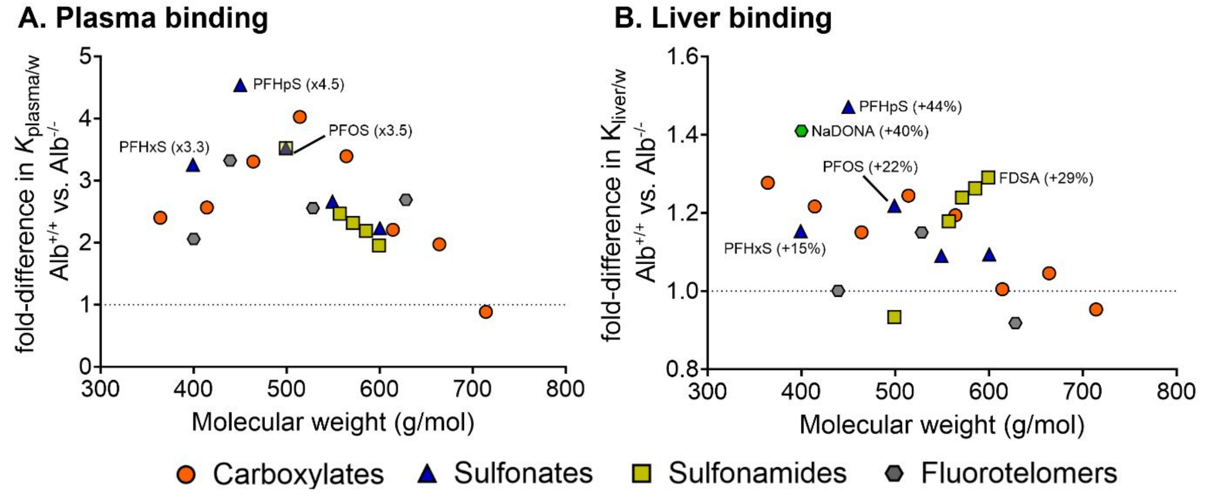

Figure S5. Fold-difference in plasma-water (A.) and liver-water (B.) partition coefficients for tissues sampled from Alb$^{-/-}$ and Alb$^{+/+}$ mice (n=3 per genotype and tissue). Please see section 2.6 of the main manuscript for method details. PFAS classes are indicated by the color and shape of symbols with orange circles for carboxylates, blue triangles for sulfonates, yellow squares for sulfonamides, and grey hexagons for fluorotelomers. Individual PFAS are highlighted by their abbreviations. The dotted line shows a ratio of 1.

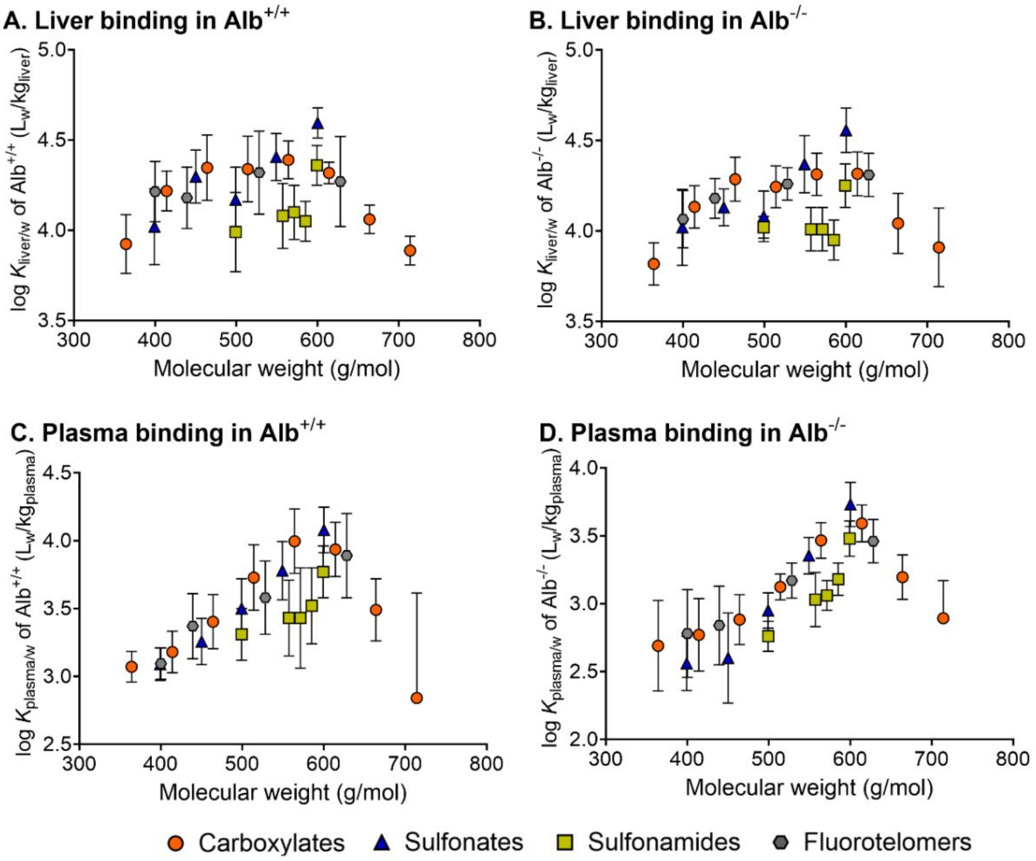

Figure S6. Plasma-water and liver-water partition coefficients as measured by C18 fiber-based binding experiments using livers (A. and B.) and plasma (C. and D.) from vehicle-control $Alb^{+/+}$ (A. and C.) and $Alb^{-/-}$ (B. and D.) mice (n=3 per genotype). Please see section 2.6 of the main manuscript for method details. PFAS classes are indicated by the color and shape of symbols with orange circles for carboxylates, blue triangles for sulfonates, yellow squares for sulfonamides, and grey hexagons for fluorotelomers.

S-6. Temporal changes in body weights.

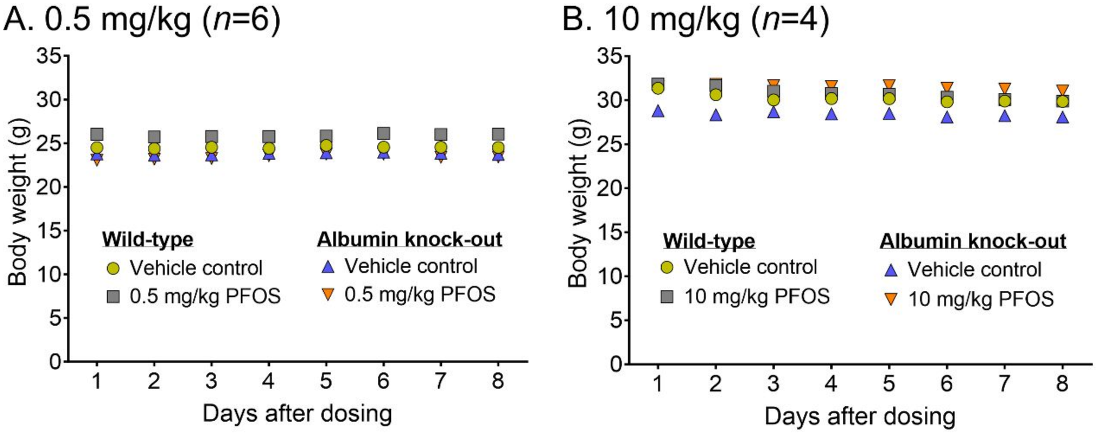

Figure S7. Body weights (grams) measured over 8 days of wild-type ($Alb^{+/+}$) and albumin knock-out ($Alb^{-/-}$) mice that were dosed with a vehicle control or with 0.5 mg/kg_{bw} (A.) or 10 mg/kg_{bw} (B.) dose of PFOS.

S-7. Changes in lipid metabolism gene expression.

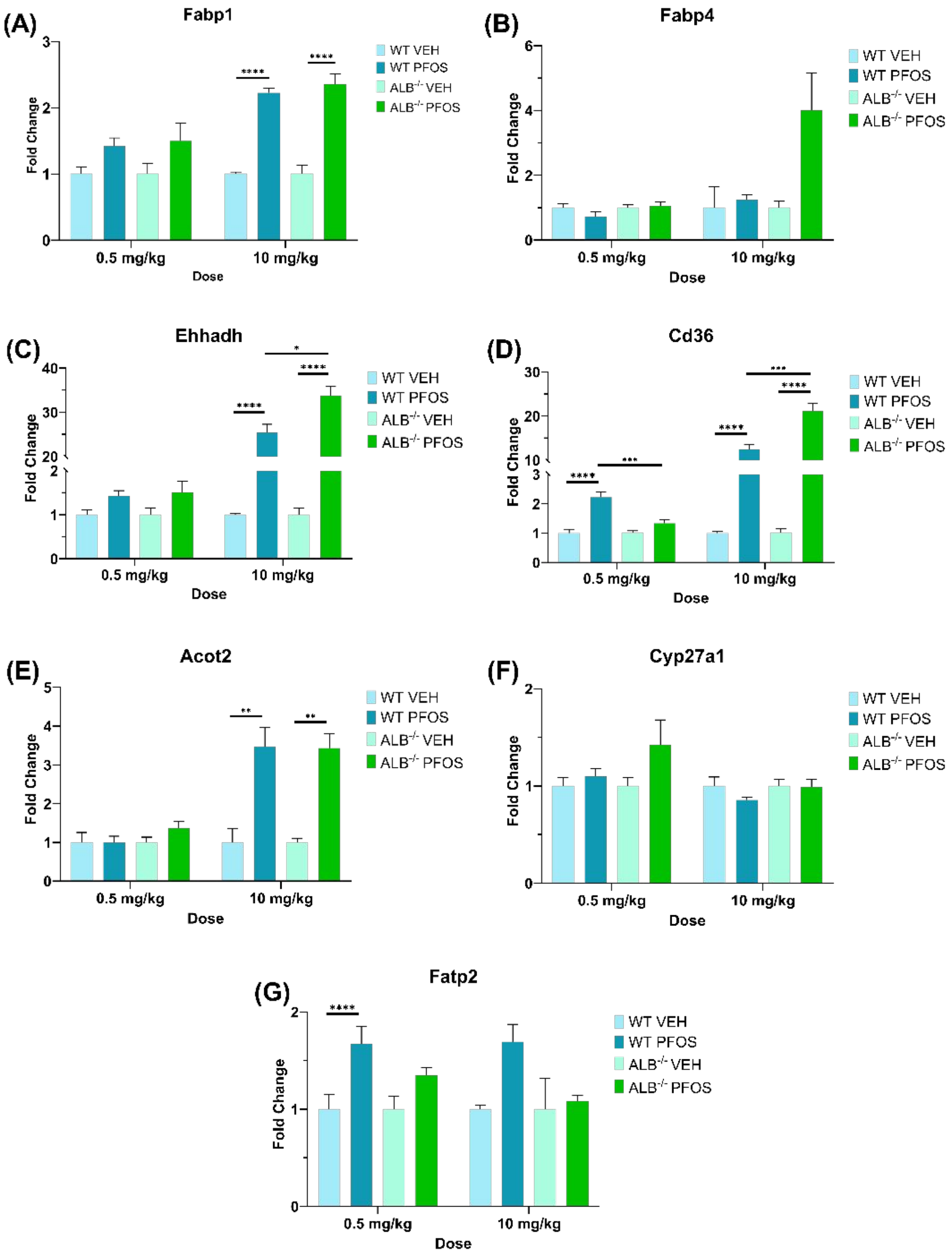

Figure S8. Lipid metabolism and accumulation gene expression changes in livers dosed daily with either 0.5 mg/kg-bw/day or 10 mg/kg-bw/day PFOS. Treatment groups included: WT ($Alb^{+/+}$) VEH (0.5% Tween 20), WT ($Alb^{+/+}$) PFOS (0.5 or 10 mg/kg), $Alb^{-/-}$ VEH (0.5% Tween 20), $Alb^{-/-}$ PFOS (0.5 or 10 mg/kg). The 0.5 mg/kg dosed study had and n = 6/treatment group, the 10 mg/kg dosed study had and n = 4/treatment group. (A) Fabp1, (B) Fabp4, (C) Ehhadh, (D) Ced36, (E) Acot2, (F) Cyp27a1, (G) Fatp2. Fold change is shown as mean ± standard error (SEM). Data was statistically analyzed using a two-way mixed analysis of variance (ANOVA) followed by a Šídák post-hoc test. Significant differences among the multiple comparisons are highlighted with asterisks.

S-8. Altered liver pathways.

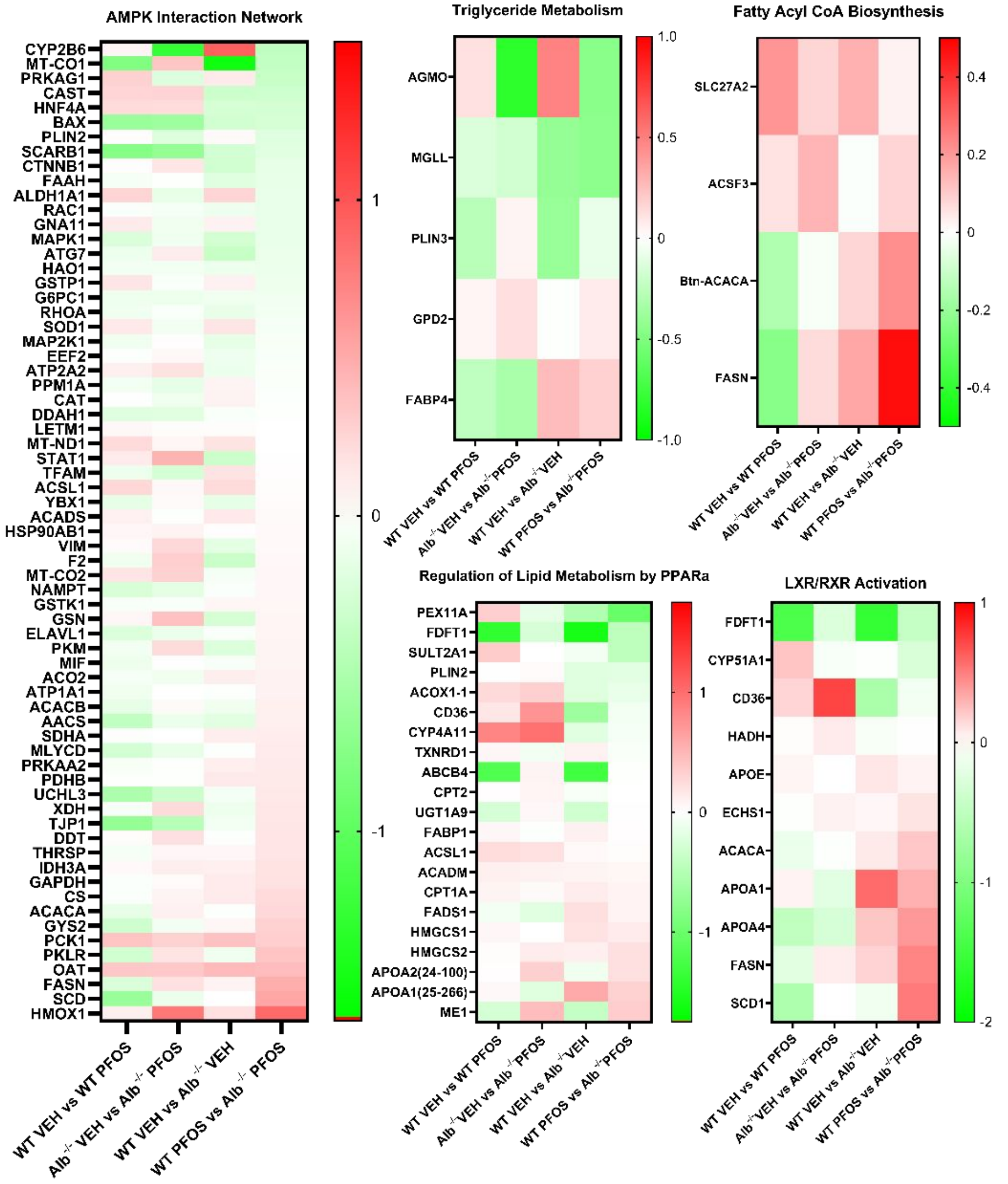

Figure S9. Heat maps of altered liver pathways in response to PFOS exposure in WT ($Alb^{+/+}$) and $Alb^{-/-}$ mice. Heat maps were generated from canonical pathway data extracted from IPA; log2FC data represents the up/downregulation of proteins, p-value is not necessarily significant. Pathways included: the AMPK interaction network, triglyceride metabolism, fatty acyl coA biosynthesis, the regulation of lipid metabolism by PPARa, and LXR/RXR activation.

S-9. Protein interaction networks.

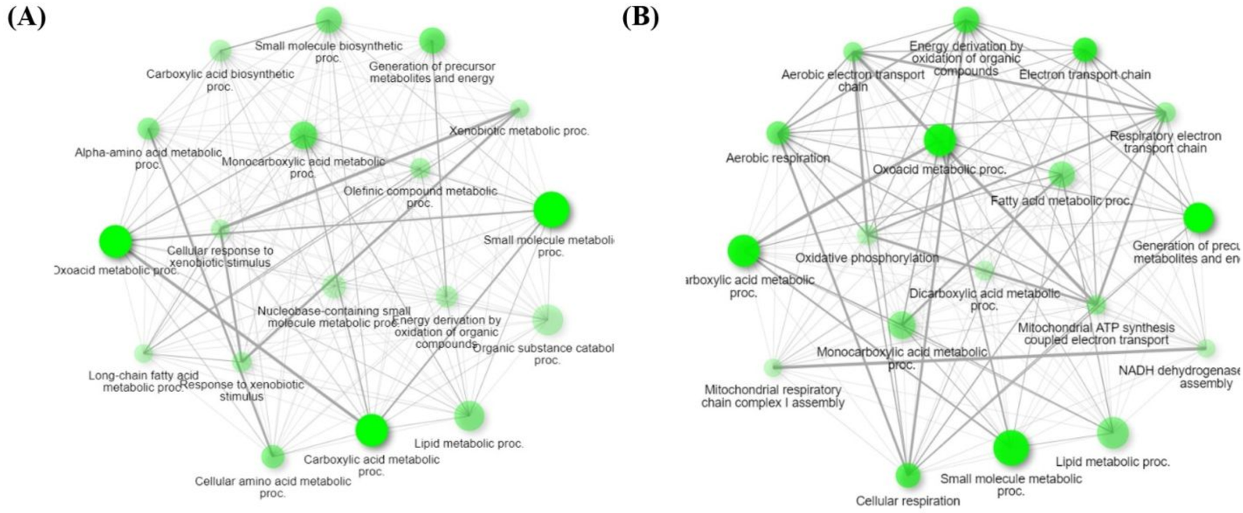

Figure S10. Interaction networks of proteins involved in biological processes unique to WT (Alb$^{+/+}$, A.) and Alb$^{-/-}$ mice (B.) exposed to PFOS. Interaction networks were created by Log2 transforming the treatment comparison fold changes and filtering out all insignificant Log2FC values ($p < 0.05$). Data was loaded into ShinyGO and interactive plots were created to reveal relationships between enriched pathways. Pathways (nodes) are connected if they share ≥20% of proteins. The shade of the node indicates significantly enriched proteins; node size indicates proteins set size; and line thickness indicates the number of overlapped proteins.
